# Test-Retest Reliability of Innovated Strength Tests for Hip Muscles

**DOI:** 10.1371/journal.pone.0081149

**Published:** 2013-11-19

**Authors:** Christophe Meyer, Kristoff Corten, Mariska Wesseling, Koen Peers, Jean-Pierre Simon, Ilse Jonkers, Kaat Desloovere

**Affiliations:** 1 KU Leuven Department of Rehabilitation Sciences, Faculty of Kinesiology and Rehabilitation Sciences, Leuven, Belgium; 2 UZ Pellenberg Orthopedic Department, University Hospitals Leuven, Pellenberg, Belgium; 3 KU Leuven Department of Human Movement Biomechanics, Faculty of Kinesiology and Rehabilitation Sciences, Leuven, Belgium; 4 UZ Pellenberg, Department of Physical Medicine and Rehabilitation, University Hospitals Leuven, Pellenberg, Belgium; 5 KU Leuven Department of Development and Regeneration, Faculty of Medicine, Leuven, Belgium; University of Utah, United States of America

## Abstract

The burden of hip muscles weakness and its relation to other impairments has been well documented. It is therefore a pre-requisite to have a reliable method for clinical assessment of hip muscles function allowing the design and implementation of a proper strengthening program. Motor-driven dynamometry has been widely accepted as the gold-standard for lower limb muscle strength assessment but is mainly related to the knee joint. Studies focusing on the hip joint are less exhaustive and somewhat discrepant with regard to optimal participants position, consequently influencing outcome measures. Thus, we aimed to develop a standardized test setup for the assessment of hip muscles strength, i.e. flexors/extensors and abductors/adductors, with improved participant stability and to define its psychometric characteristics. Eighteen participants performed unilateral isokinetic and isometric contractions of the hip muscles in the sagittal and coronal plane at two separate occasions. Peak torque and normalized peak torque were measured for each contraction. Relative and absolute measures of reliability were calculated using the intraclass correlation coefficient and standard error of measurement, respectively. Results from this study revealed higher levels of between-day reliability of isokinetic/isometric hip abduction/flexion peak torque compared to existing literature. The least reliable measures were found for hip extension and adduction, which could be explained by a less efficient stabilization technique. Our study additionally provided a first set of reference normalized data which can be used in future research.

## Introduction

Hip muscles play an important role in the normal function of the lower limb [1,2]. Literature shows that hip muscle dysfunction is associated with low back pain [3,4] and other lower limb impairments or diseases such as patellofemoral pain syndrome [5–7] and hip and knee osteoarthritis [8,9]. Such dysfunction can either be inherent to an underlying pathology or a secondary consequence of pathology or surgical intervention, e.g. total hip arthroplasty (THA) [10,11]. Several gait studies showed that patients following THA present an abnormal gait pattern related to iatrogenic gluteal muscle weakness [12,13]. These findings point towards the importance of an adequate assessment of muscle function allowing the design and implementation of a proper strengthening program.

The assessment of muscle strength can be done using several testing methods such as: manual muscle testing, hand held dynamometry (HHD) and motor-driven dynamometry. The first one, rating muscle strength from 0 to 5, is considered a subjective tool with poor quantitative precision [14]. The other two methods are more objective tools. HHD has the advantage of being user-friendly and inexpensive with an established reliability in several populations [15–18] and muscle groups [19–21]. However, a major drawback of HHD entails the lack of standardization of the participants’ starting position and the placement of the assessor and the dynamometer.

Consequently, motor-driven dynamometry is still considered the gold-standard with a thorough standardization and the results are not influenced by a strength imbalance between the participant and the assessor. Moreover, it allows performing both isometric and isokinetic testing. Although, isometric testing is shown to be a reliable and valid method [22,23], isokinetic testing is more representative of muscle action during dynamic tasks of daily life. Despite no actions in real life occur at constant velocity, isokinetic testing provides a more natural movement condition due its dynamic nature, whereby a maximal torque can be generated throughout the whole range of motion.

So far, lower limb muscle strength tests have mainly focused on the assessment of the knee joint [24–26]. Few studies have been dedicated to the hip joint, for which testing position, range of motion, and stabilization techniques have varied and therefore lead to discrepancies regarding reliability measures of hip torque generation. Julia et al. [27] reported intraclass correlation coefficient (ICC) values of 0.94 for concentric hip flexion at 60°/s, tested in supine position, whereas Arokoski et al. [9] reported values of only 0.7. These two studies also showed dissimilar findings for hip extension, which could be explained by a varying tested range of motion. On the other hand, although Arokoski et al. [9] and Claiborne et al. [28] used different methodologies for testing isometric hip abduction, i.e. standing versus supine, they found comparable results. Unlike this latter comparison, Widler et al. [23] compared various positions for isometric hip strength measures and reported side-lying as the most valid and reliable method. Such divergence in results and conclusions between studies emphasize the need for standardization of testing protocols in order to target specific muscles and inhibit compensatory movements during strength measurements. 

Therefore, this study aimed at improving reliability of hip muscle strength evaluations using dynamometer experiments by developing an innovative standardized test setup providing optimal patient stability. Additionally, this study evaluates the reliability of these innovative hip strength tests.

## Methods

### Participants

**Figure 1:**
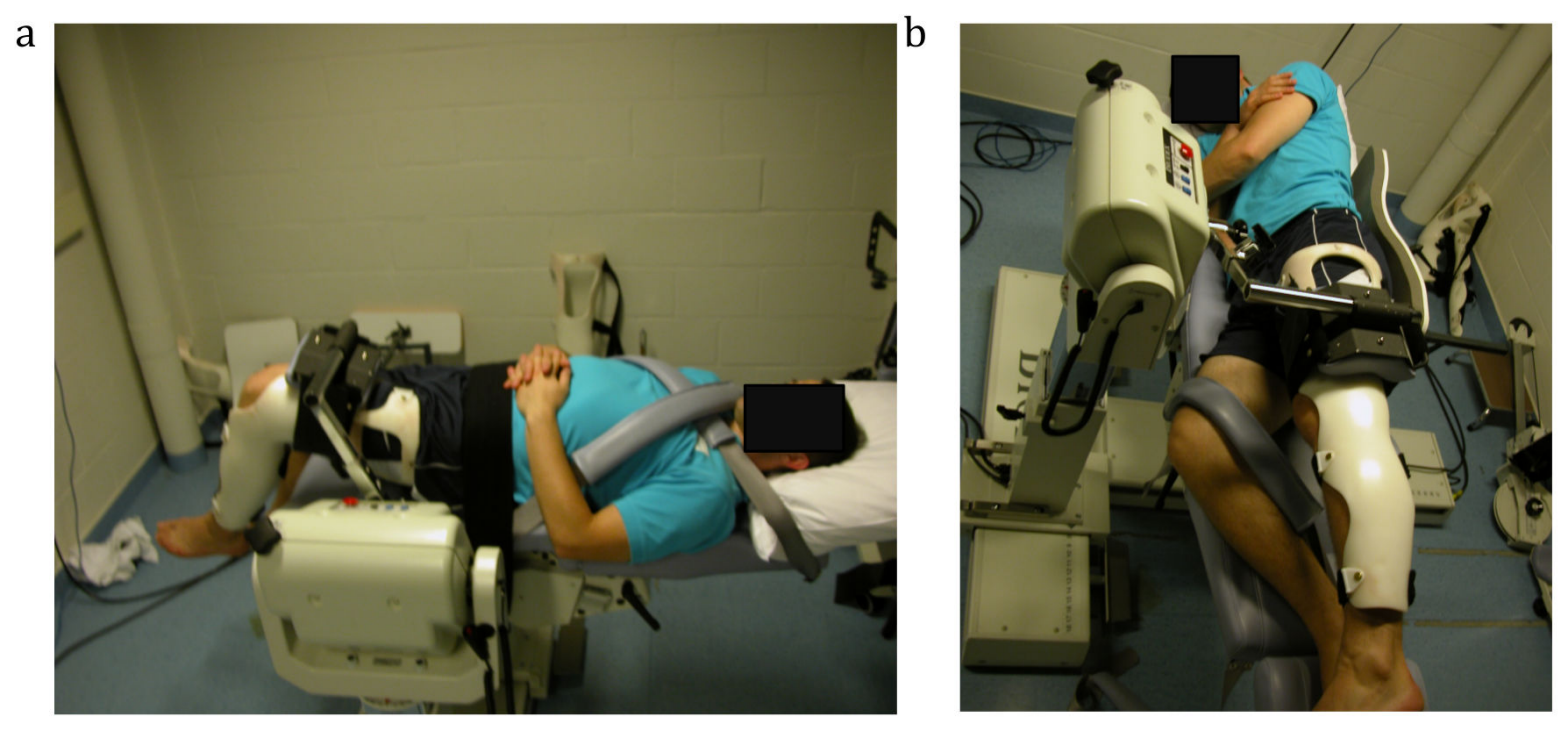
Protocol setup. a) On the left hand side, hip flexion setup and b) on the right hand side, hip abduction setup.

A total of 10 men and 8 women volunteered to participate in this study after signing an informed consent. Also, written informed consent (as outlined in PLOS consent form) was obtained from the individual presented in Figure 1 of this manuscript to publication of his photographs. Participants were excluded in case of low back pain, lower limb muscle pathology and/or joint degeneration, cardiovascular, metabolic or pulmonary disease, or a body mass index (BMI) > 30 kg/m^2^. Mean (±SD) age, height, weight and BMI of the participants were 44 years (±12.1), 1.75m (±0.10), 74.97 kg (±10.92) and 24.44kg/m^2^ (±2.88), respectively. The study was approved by the local Ethics Committee (‘‘Commissie Medische Ethiek van de Universitaire Ziekenhuizen Leuven’’) and conducted in accordance with the declaration of Helsinki.

### Study design

To investigate test-retest reliability of the developed protocol to assess muscle strength, two identical test sessions were performed one week apart at the same time of the day. The same investigator performed all measurements and assured an identical sequence of exercises and setups. Maximal isokinetic and isometric peak torques were assessed after a warm-up period of 10 minutes on a cycle ergometer at a low pre-set intensity. Participants were verbally encouraged to ensure a maximal effort during all trials. 

### Setup and exercises protocols

Muscle strength tests were performed for the hip joint using the Biodex isokinetic dynamometer, whereby hip abduction/adduction and hip flexion/extension of the non-dominant leg were randomly tested. Isokinetic testing was always performed prior to isometric testing for all joint movements. To get accustomed to the test procedure, participants were first asked to perform three submaximal repetitions at the same speed as during the actual protocol. Next, they had to perform three isokinetic concentric/concentric contractions at a low velocity (60°/s) and five at a high velocity (120°/s). Between both velocity conditions, there was a rest period of one minute. After isokinetic testing, participants had to perform three sustained maximal voluntary isometric contractions (MVIC) of 6 seconds with a 30 seconds rest period in between. Participants were also given an appropriate rest period between each different tested muscle group (up to 5 minutes). 

Prior to performing the isokinetic and isometric tests, participants were fixed in a standardized position. For the hip abduction/adduction test, we developed an innovative setup to maximize participants’ stability and minimize compensatory mechanisms (Figure 1a). Contrary to the manufacturer's instructions, participants were positioned on their side on the dynamometer chair facing the dynamometer, therefore giving less space for compensatory movements. This position provided a backrest and allowed participants to hold the handhold in front of them, thereby minimizing trunk and pelvic rotation during testing. The tested leg was secured into a brace to ensure full knee extension and was strapped to the dynamometer pad at the femur level to avoid hip rotation. The starting position for the hip joint was set at 0° of flexion and at full adduction during isokinetic testing whereas it was set at 10° of abduction for isometric testing. The non-tested hip and knee were flexed (45° and 60°, respectively) for comfort and stabilization and strapped to the dynamometer chair. 

For the hip flexion/extension test, participants laid supine on the dynamometer chair with the chair back completely flattened (Figure 1b). The tested hip was at 0° of flexion, with 90° of knee flexion, and secured into a brace. However for isometric testing, it was set at 45° of hip flexion. The brace was used to standardize the tensor fascia latae and rectus femoris muscle length during hip flexion. The tested thigh was strapped to the dynamometer pad at femur level. The non-tested thigh was strapped to the dynamometer chair (0° of hip flexion), with the shank not fixed. The pelvis and trunk were secured to the dynamometer chair with straps. For each setup, prior to testing, the mass of the tested limb was measured in relaxed position by the isokinetic device to allow correction for gravity.

For both tests, the strap around the tested leg was positioned proximal to the knee joint, at 75 percent of the femur length. Femur length was measured from the most prominent palpable site of the greater trochanter until the lateral condyle of the femur. Furthermore, the dynamometer height and chair and dynamometer fore-aft distances were adjusted to ensure that the dynamometer pivot corresponded to the greater trochanter level. These dimensions were kept the same for the two test sessions. Lastly, complete range of motion was assessed for each isokinetic test. 

### Parameter extraction and data analysis

Peak torque (PT) and normalized peak torque (PT _norm_) as reported by Bazett-Jones et al. [29] were selected as outcome parameters. The maximum value during each set of repetitions was retained and used for statistical analysis. Mean and SD were calculated for the maximum PT and PT_norm_. A Wilcoxon signed rank test was used to analyze the differences between the two test sessions, with significance level set at 0.05. Test-retest reliability of the isokinetic and isometric PT and PT_norm_ was assessed via the intraclass correlation coefficient (ICC_2,1_) [30]. ICC values < 0.8 were considered "insufficient", between 0.8 and 0.9 "moderate", and ICC values > 0.9 "high" [31]. The standard error of measurement (SEM) was calculated to determine absolute reliability, using the square root of the mean squared error obtained from the 2-way ANOVA [30], and expressed as a percentage of the mean. The between subject coefficient of variation (CV_*b*_) was also calculated using the square root of the variance component estimate from the 2-way ANOVA divided by the overall mean. Data analysis was conducted with Microsoft Excel (Microsoft Excel for Mac 2011) using a custom-made macro and the XLSTAT package for excel. Primary data as well the Excel macro are available on request.

## Results

None of the participants dropped out. However, due to technical issues, only 17 and 15 out of 18 tests-retests could be analyzed for isometric hip flexion and extension, respectively. None of the participants were experienced with dynamometry prior to the study and none of them reported any discomfort and/or pain during or after testing sessions.

Tables 1 and 2 respectively summarize the between day mean peak torque and normalized peak torque values with their respective 95% CI and the errors of measurements.

**Table 1:** Reliability, variability and clinical important change of hip torque measures using Biodex dynamometer.

**Test condition**	**Hip tests**	**Mean (95% CI)**		**ICC (95%CI)**		**SEM**	**SEM (%)**	**SRD_95_**	**SRD_95_ (%)**	**CV _*b*_ (%)**
**Isometric**	Abduction	117.40	101.53-133.40	0.91	0.77-0.96	10.12	8.62	28.06	23.89	27.11
	Flexion	103.79	85.83-121.75	0.97	0.93-0.99	4.47	4.31	12.40	11.94	34.10
	Extension	161.10	142.50-179.70	0.77	0.43-0.91	15.88	9.86	44.02	27.32	19.97
**Isokinetic**	Abduction	120.23	104.47-136.00	0.83	0.60-0.93	13.09	10.89	36.28	30.17	25.62
**60°/s**	Adduction	91.55	74.11-108.99	0.68	0.33-0.87	15.61	17.05	43.28	47.27	35.34
	Flexion	122.66	104.28-141.04	0.92	0.80-0.97	10.51	8.57	29.13	23.75	30.06
	Extension	130.50	107.97-153.03	0.84	0.61-0.93	12.66	9.70	35.10	26.90	33.73
**Isokinetic**	Abduction	106.24	90.71-121.78	0.89	0.74-0.96	10.53	9.91	29.19	27.48	29.12
**120°/s**	Adduction	85.15	70.76-99.54	0.76	0.46-0.90	12.02	14.12	33.32	39.13	32.24
	Flexion	105.18	89.28-121.08	0.93	0.82-0.97	8.41	8.00	23.32	22.17	30.39
	Extension	123.30	101.92-144.68	0.80	0.55-0.92	16.11	13.06	44.65	36.21	33.58

**Table 2:** Reliability, variability and clinical important change of normalized hip torque measures using Biodex dynamometer.

**Test condition**	**Hip tests**	**Mean (95% CI)**		**ICC (95%CI)**		**SEM**	**SEM (%)**	**SRD_95_**	**SRD_95_ (%)**	**CV _*b*_ (%)**
**Isometric**	Abduction	7.71	5.68-9.75	0.96	0.91-0.99	0.80	10.41	2.23	28.86	53.66
	Flexion	6.17	4.57-7.77	0.98	0.95-0.99	0.37	6.04	1.04	16.79	49.12
	Extension	11.54	7.80-15.28	0.98	0.95-0.99	0.96	8.28	2.65	22.95	59.65
**Isokinetic**	Abduction	8.00	5.85-10.15	0.97	0.91-0.99	0.76	9.48	2.10	26.26	54.65
**60°/s**	Adduction	5.81	4.13-7.50	0.79	0.52-0.91	1.38	23.79	3.83	65.96	55.85
	Flexion	7.87	5.88-9.86	0.99	0.96-0.99	0.48	6.09	1.33	16.89	51.62
	Extension	8.8	5.97-11.63	0.93	0.81-0.97	1.18	13.37	3.26	37.07	64.54
**Isokinetic**	Abduction	7.00	5.13-8.87	0.96	0.90-0.99	0.76	10.85	2.11	30.07	54.23
**120°/s**	Adduction	5.58	3.94-7.23	0.93	0.83-0.97	0.74	13.28	2.05	36.81	59.24
	Flexion	6.75	5-8.49	0.98	0.95-0.99	0.47	7.00	1.31	19.41	52.76
	Extension	8.16	5.76-10.55	0.96	0.91-0.99	0.88	10.74	2.43	29.77	62.97

Abbreviations: CI, confidence intervals; Nm, Newton meter; Nm/kg, newton meter per kilogram; s, second; ICC, intraclass correlation coefficient; SEM, standard error of measurement; SRD_95_, smallest real difference at 95% confidence interval; CV_b_, between subject variability.

**Figure 2:**
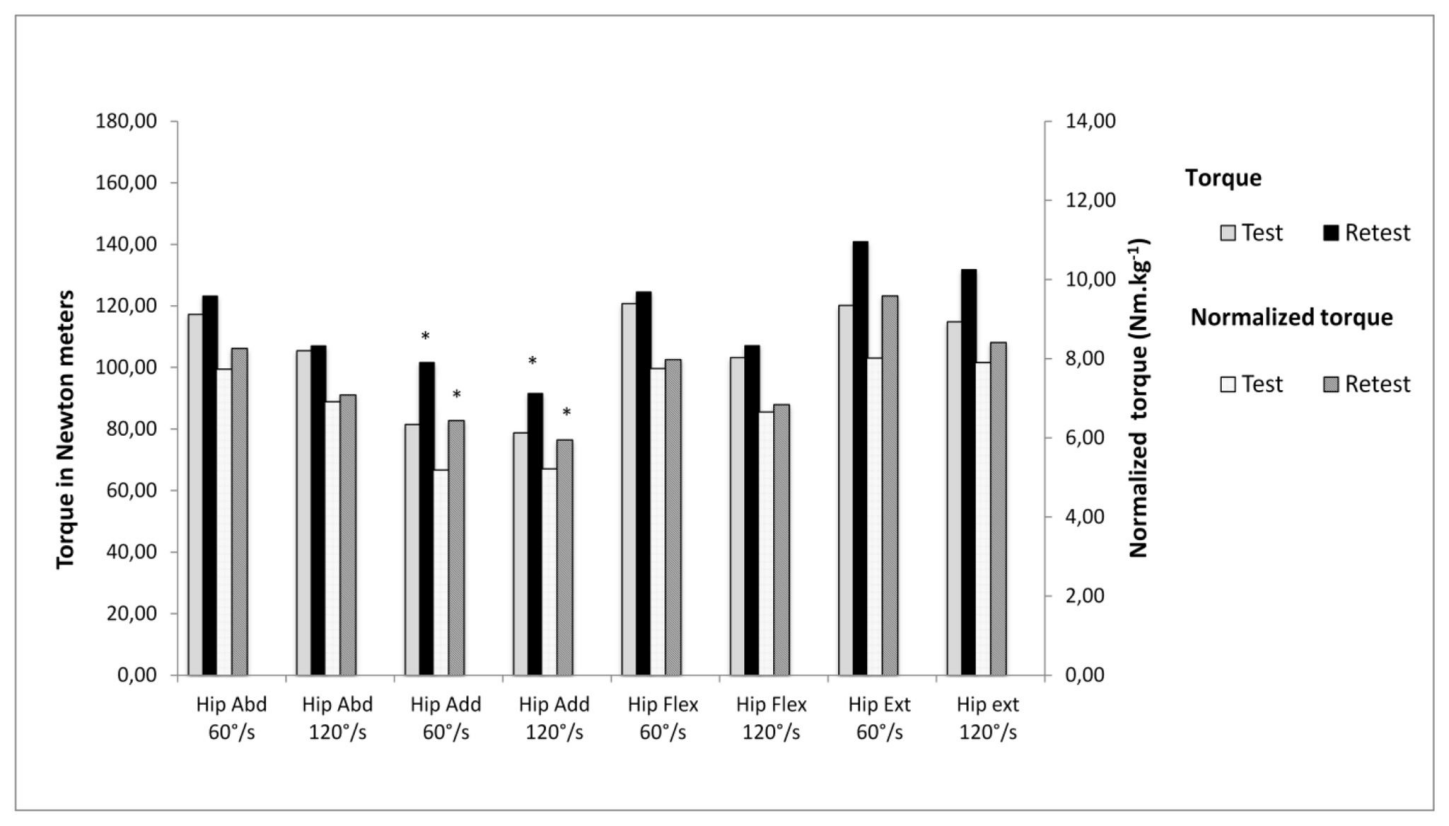
Mean peak test-retest of isokinetic peak torque expressed in relative and normalized value. *p<0.05, retest significantly different from test using Wilcoxon signed rank test.

Figure 2 summarizes the mean peak torque and mean normalized peak torque during isokinetic hip abduction, adduction, flexion and extension at 60 and 120°/s for the two testing days. Mean peak values for both velocities and all conditions were found to be higher during the second testing day. Statistical differences between day 1 and day 2 were found for isokinetic hip adduction and extension at both velocities.

**Figure 3:**
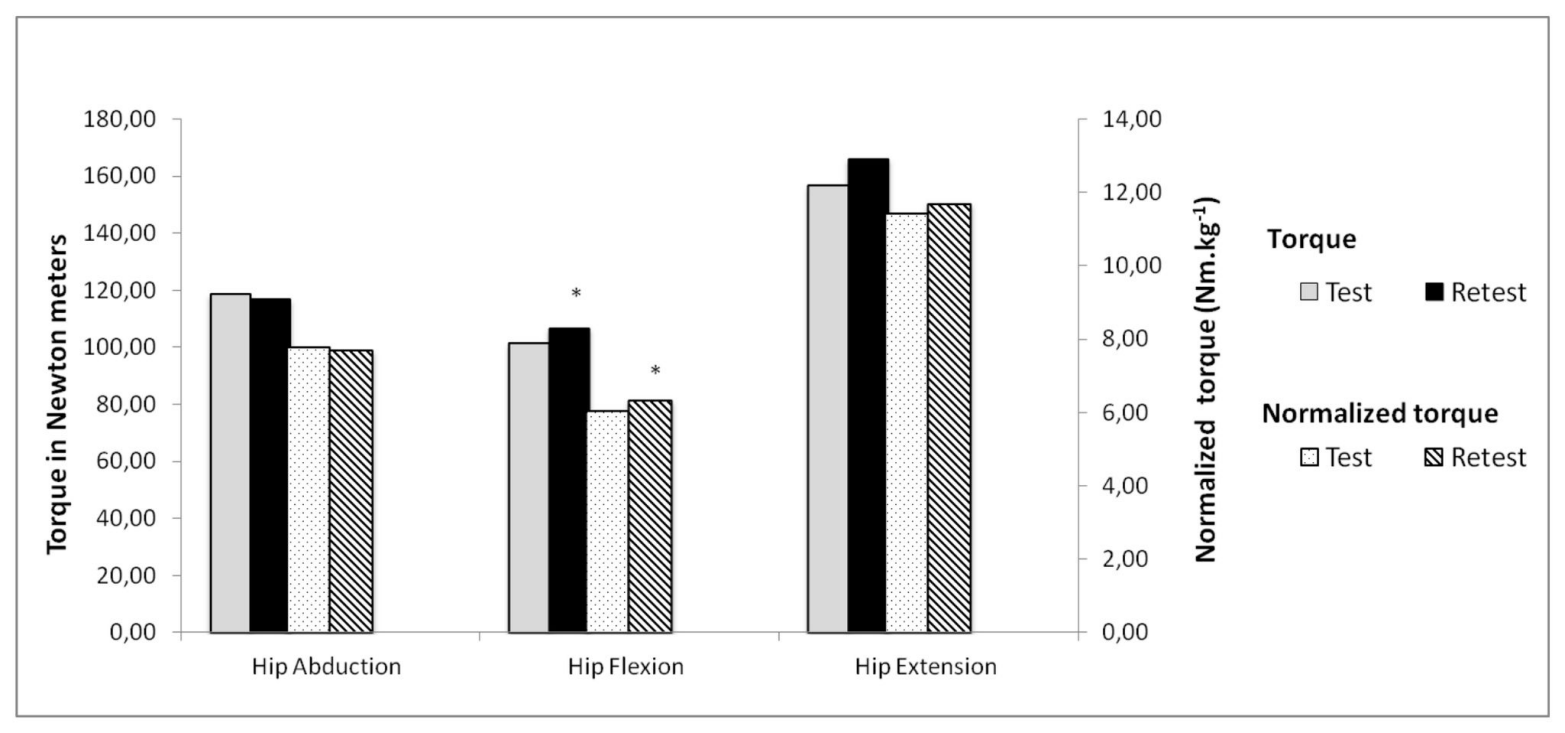
Mean peak test-retest of isometric peak torque expressed in relative and normalized value. *p<0.05, retest significantly different from test using Wilcoxon signed rank test.

Figure 3 shows the mean peak torque and mean normalized peak torque during isometric hip abduction, flexion and extension. Contrary to isokinetic testing, values for day 2 were not always found to be higher than day 1. Only for isometric hip flexion significant difference was found from day 2 compared with day 1. 

**Figure 4:**
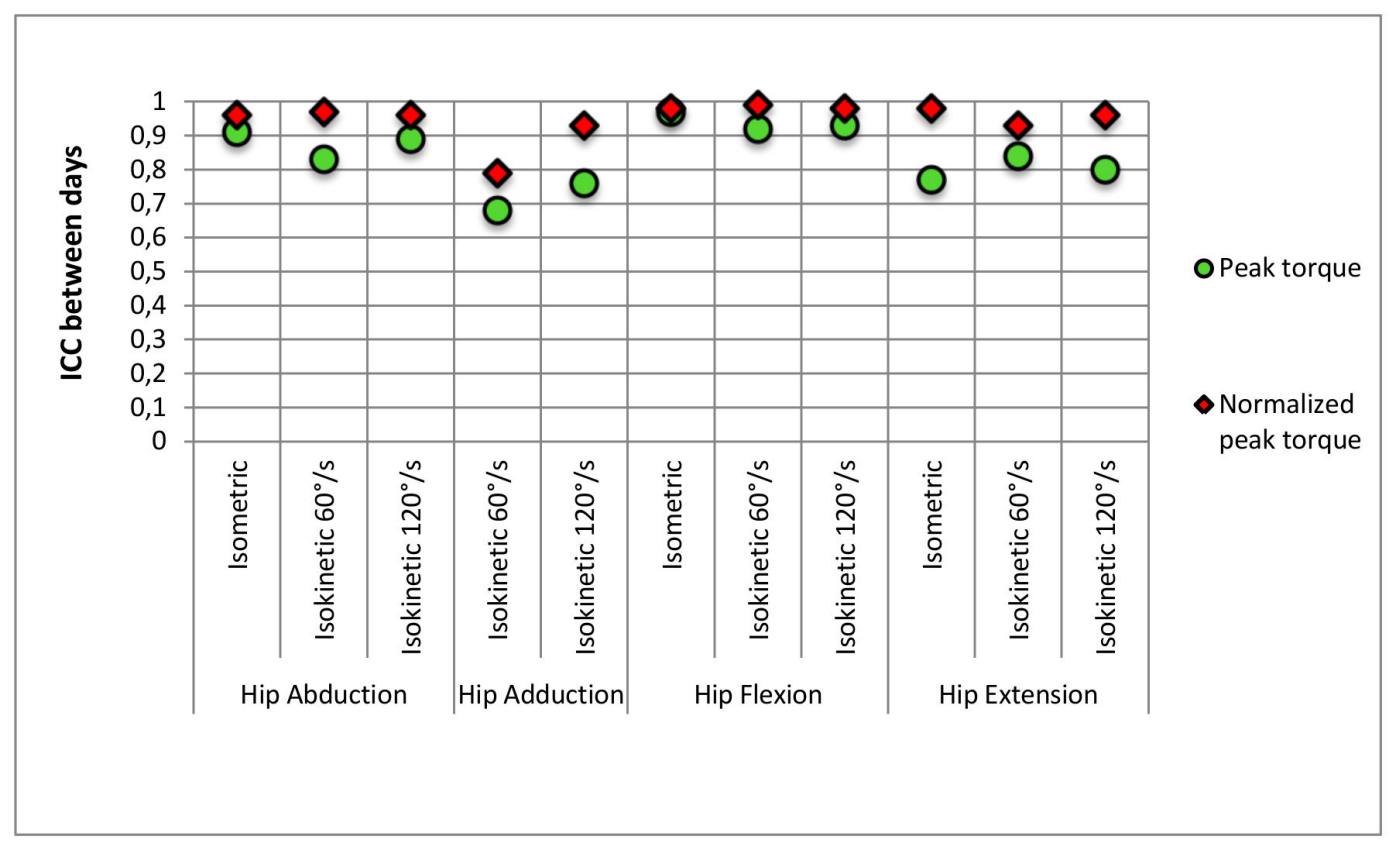
Reliability of isokinetic and isometric tests.

Figure 4 represents the between days ICCs for each test and condition testing. When data were expressed as absolute values, except for isokinetic hip adduction and isometric hip extension, ICCs were found to be moderate to high (0.80 ≤ ICC ≤ 0.97) with the highest value being for isometric hip flexion. Moreover, when data were normalized, all ICCs were found to be high (0.93 ≤ ICC ≤ 0.98) apart from isokinetic hip adduction at 60°/s (ICC = 0.79).

## Discussion

The aim of this study was to develop a new hip strength measurement protocol using the Biodex isokinetic dynamometer with the purpose of minimizing compensatory mechanisms and evaluate whether reliability results would be enhanced when compared with available literature. The reason for improving hip muscle strength tests remains in the difficulty to voluntarily achieve isolated hip muscle group recruitment for the specific positions tested in the present study. As literature corroborated hip muscle weakness morbidity, it is of paramount importance to properly measure these muscle groups. However, reference studies of hip muscle strength assessment are discrepant regarding protocols and setups and little consensus has been achieved. 

With varying testing protocols and setups, available ICC ranged from 0.04 to 0.94 [9,32] for torque measures of the hip flexors/extensors and abductors/adductors. Our ICC values were mostly found to be between moderate and high when reported as absolute peak torque values (0.68 ≤ ICC ≤ 0.97). Isometric hip abduction and isometric hip flexion were found to be highly reliable (ICC > 0.9). Generally, hip adduction and isokinetic hip extension demonstrated the lowest ICCs, confirmed by a significant difference between day 1 and day 2 (Figure 2). We found higher ICC values for PT of isokinetic hip flexion at 60°/s (0.92) compared to Claiborne et al.[28]. These authors generally reported lower ICC values than the current study. However they measured in a standing position whereas we conducted the test in lying position, which is the predominant test position used in available literature [9,27]. While Julia et al.[27] found comparable results for isokinetic hip flexion (ICC > 0.90), Emery et al.[32] reported an extremely low ICC (0.04). One of the explanations of the discrepancies with our results could lie in the different testing position, more specifically in the optimized stabilization technique we introduced. The use of the brace allowed us to only measure hip flexion torque since movement of the shank segment was restricted. We believe that such a brace restricting leg extension combined with straps around pelvis and contralateral leg is innovative and helps in minimizing compensatory mechanism as well as isolates hip flexors activity.

Regarding isometric hip abduction, we found an ICC value of 0.91 (PT) which is relatively higher than the one reported by Arokoski et al. [9] (ICC=0.84) who measured torque in a supine position. However, comparing results for hip abduction in side-lying position from our study to results from other studies is rather difficult since testing position has been under debate and only few studies were dedicated to side-lying testing [28,29,32]. While Widler et al. [23] recommended side-lying positions as the most valid and reliable method for hip abductors strength assessment using HHD, we decided to apply it in isokinetic settings that would allow us to report detailed innovative measurement technique and reliability results. Thorborg et al. [19] reported an ICC of 0.76 for hip peak abduction torque. However, they performed the measurements with a hand-held dynamometer, which could have compromised the participant's stability and thus causing their lower reliability. This latter issue was the main focus of our study protocol and therefore we aimed to improve stability of participants by adding a fixed backboard to the dynamometer rails whereby, pelvic rotation would be minimized. We additionally asked participants to lay at the edge of the seat, therefore reducing mobility. Based on our observations, we assume that the combination of the backboard and the position on the table explains our high reliability results since it reduces pelvic rotation and hip flexion. To our knowledge, only the study of Laheru et al. [33] addressed the issue of optimizing hip abduction testing but demonstrated no correlation between reduced pelvic rotation and enhanced reliability measures which does not confirm our observations. Nevertheless, their low peak torque values, half of ours, highlighted the need for further studies.

In general, our results showed moderate to high reliability (ICC > 0.7) along with low SEM values. The SEM values for all measurements ranged from 4.31% to 17.05% (4.47Nm to 15.61Nm) for PT and from 6.04% to 23.79% (0.37Nm.kg^-1^ to 1.38 Nm.kg^-1^) for PT_norm_. These values concur with the ones reported by Claiborne et al.[28], except for PT of isokinetic hip adduction for which they had an equivalent SEM value up to 24.11Nm while we had only 15.61Nm. Generally, hip flexion followed by hip abduction had lowest SEM values for both isokinetic and isometric strength tests (SEM < 13.1%). Low normalized SEM values were found, in the range of 3–11% for PT and 6-11% for PT_norm_, except for isokinetic hip adduction and extension which indicates that such measures are accurate and suitable for clinical evaluations.

Finally, this study also reports novel normalized data establishing a starting point for a new reference hip strength dataset. However, its clinical validity and implementation would require a larger sample size. Until now, several methods have been applied [34]. However, when using body mass, it is assumed that torque is proportional to body mass, which leads to misrepresentative findings. The recent study of Bazett-Jones et al. [29] reported allometric scaling values, meant to remove body-size effect for hip muscle strength tests. They reported a gender specific scaling value that has been shown to be valid and reliable. We hence reported peak torque values as well as reliability results using their normalization technique for providing the first reference control dataset using this independent body mass normalization method. It can be observed that ICC scores improve when normalizing peak torque data. These higher ICC values result from an increased between subjects variability (CV**_b_**). However, ICC by itself is not sufficient to report reliability since its magnitude is largely influenced by the between-subjects variability, therefore, we also reported the concordant SEM values. While comparing the SEM values between our non-normalized and normalized values (Tables 1 and 2), one notices a non-linear relationship between the order of magnitude change in ICC and SEM. Although data are normalized, the SEM values remain similar (mostly < 11%).

### Study limitations

The testing order may have influenced results. We decided to always perform the isokinetic strength test before the isometric test, which could have affected the maximal isometric force the participant could generate because of muscles fatigue. However, due to the interval rest phases, we were convinced that this testing order had minor impact on the outcome and therefore the order was kept the same in both test sessions. Other factors influencing the test reliability relate to the participant’s motivational status and the test setup. Although the motivational factor cannot be completely controlled, the investigator assuring equivalent motivation provided similar encouragement to all test participants. To maximize standardization and minimize compensatory movements, we introduced an innovative setup whereby we braced the tested leg, used pelvis and trunk stabilization straps and a backrest. However, for hip adduction and extension, we still observed less reliable results as shown by low ICC and high SEM values. This might have been due to a less effective stabilization and compensatory movement of the pelvis. Nonetheless, only one test, hip adduction (ICC ≤ 0.79), was found to be insufficient for PT and PT_norm_. Lastly, we investigated the hip of the non-dominant side whereas most studies investigated both sides. This could be considered as a drawback as one might expect poorer reliability than the dominant side. Nevertheless, we found high ICCs with concordant low SEM values for the non-dominant side, ensuring a good reliability of the strength test and the choice of this side ensures a more reasonable link to evaluations in pathological population.

## Conclusions

The current study showed moderate to high reliability results for the innovated protocol to assess hip muscle strength in a standardized way for major hip muscles. We also provided novel normalized data that can be further used for population comparison. However, although adduction and extension were found to be more reliable than existing literature, they remain the least reliable and therefore imply further development to improve subject’s stability and avoid compensatory mechanisms at the pelvis level. Future work implies involvement of a patient population. 
